# Lipid-Induced Insulin Resistance in Skeletal Muscle: The Chase for the Culprit Goes from Total Intramuscular Fat to Lipid Intermediates, and Finally to Species of Lipid Intermediates

**DOI:** 10.3390/nu8080466

**Published:** 2016-07-29

**Authors:** Soressa M. Kitessa, Mahinda Y. Abeywardena

**Affiliations:** 1 CSIRO Health and Biosecurity, Kintore Avenue, Adelaide 5000, SA, Australia; Mahinda.Abeywardena@csiro.au; 2 Division of Livestock and Farming Systems, South Australian Research and Development Institute, J S Davies Bldg, Roseworthy Campus, GPO Box 397, Adelaide 5000, SA, Australia

**Keywords:** insulin resistance, skeletal muscle, lipid intermediates, ceramides, DAG

## Abstract

The skeletal muscle is the largest organ in the body. It plays a particularly pivotal role in glucose homeostasis, as it can account for up to 40% of the body and for up to 80%–90% of insulin-stimulated glucose disposal. Hence, insulin resistance (IR) in skeletal muscle has been a focus of much research and review. The fact that skeletal muscle IR precedes β-cell dysfunction makes it an ideal target for countering the diabetes epidemic. It is generally accepted that the accumulation of lipids in the skeletal muscle, due to dietary lipid oversupply, is closely linked with IR. Our understanding of this link between intramyocellular lipids (IMCL) and glycemic control has changed over the years. Initially, skeletal muscle IR was related to total IMCL. The inconsistencies in this explanation led to the discovery that particular lipid intermediates are more important than total IMCL. The two most commonly cited lipid intermediates for causing skeletal muscle IR are ceramides and diacylglycerol (DAG) in IMCL. Still, not all cases of IR and dysfunction in glycemic control have shown an increase in either or both of these lipids. In this review, we will summarise the latest research results that, using the lipidomics approach, have elucidated DAG and ceramide species that are involved in skeletal muscle IR in animal models and human subjects.

## 1. Diabetes Epidemic and Insulin Resistance

**Figure 1:**
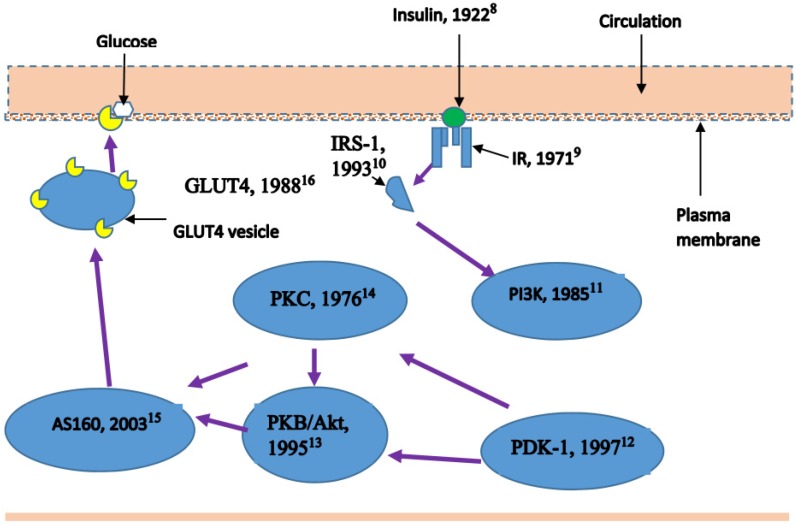
Schematic representation of the insulin signalling pathway with years of discovery of the key elements in the pathway. Numbers in superscript are reference list numbers [8,9,10,11,12,13,14,15,16].

Since the Banting group’s discovery of insulin, substantial progress has been made in elucidating the insulin signalling pathways (Figure 1). Despite such sustained breakthroughs, diabetes continues to be a global menace. It has been variously described as an epidemic or even as the plague [1] of the 21st century. It is now widely recognised that insulin resistance precedes the clinical manifestation of diabetes. The Centre for Disease Control [2] estimates that 15% to 30% of patients exhibiting prediabetes will develop Type 2 Diabetes Mellitus (T2DM) within the next five years; equivalent to 3% to 6% in any given year. Insulin resistance (IR) is a physiological state of impaired response in glucose uptake to physiological concentration of insulin. In his Banting Lecture, Reaven [3] was probably the first person to highlight IR as a common feature of human chronic diseases (prior to the evolution of the now widely accepted term-metabolic syndrome). Today, it is known that IR is strongly linked to obesity, and IR as a feature of the diseases of the metabolic syndrome is undisputed. As Samuel and Shulman [4] put it, “it is *sin qua non* with the pathogenesis of these modern diseases”. Insulin enables glucose control in two main ways: by enhancing glucose uptake in skeletal muscle and other tissues, and by inhibiting glucose production in the liver [4,5]. As the first of these two mechanisms largely involves the skeletal muscle, the importance of understating IR in skeletal muscle cannot be overstated. The seminal paper by Reaven [3] also indicated that resistance to insulin-stimulated glucose uptake was observed in 25% of non-obese individuals with normal oral glucose tolerance. Other authors also note IR as the principal feature of T2DM that can precede its clinical manifestation by 10–20 years [6]. Skeletal muscle IR is thus considered the initiating or primary defect [7] that can be detected decades ahead of the β-cell failure and hyperglycaemia. Therefore, understanding the development of IR and designing remedial strategies provides an early and cost-effective means to curb the epidemics of T2DM. This is because peripheral IR precedes β-cell dysfunction and reversing peripheral IR will arrest progression to T2DM. Before exploring the link between IMCL and IR, it is essential to briefly consider lipid transport into the skeletal muscle.

## 2. Lipid Transport into Muscle Cells

Skeletal muscle is the largest organ in the body and accounts for 80%–90% of insulin-induced glucose uptake from circulation. Muscle is considered a metabolically flexible [17] or promiscuous [18] organ because of its capacity to use both glucose and fatty acids as fuels. The flexibility of muscle fuel selection creates a particular complication in addressing ectopic lipid accumulation. This is because it is not fully understood how this selection is driven at a systemic or intramyocellular level. With respect to movement of fatty acids into muscle cells, it was initially thought that the movement of free fatty acids (FFA) from plasma into cells was through passive diffusion based on concentration gradient [19,20]. Later, saturation kinetics studies [21] pointed to the presence of fatty acid carrier proteins [22]. It is now widely recognised that there are three groups of fatty acid transporters: (A) fatty acid binding proteins (FABP); (B) fatty acid translocase (FAT), which is also known as cluster differentiation 36 (CD36); and (C) fatty acid transport proteins (FATP) [23]. The FABP are also sometimes identified based on their location: plasma membrane (FABPpm) or cytosol (FABPc). There are six main subgroups of the FATP numbered 1–6 [23]. The location and activities of each of this FATP has been elucidated using specific knockout models. A brief summary from Kazantzis and Stahl [24] is presented in Table 1.

Since its first description by Aitman et al. [25], CD36 is the most commonly studied fatty acid carrier in skeletal muscle. It is located within the cytosol of myocytes and moves back and forth to the plasma membrane (PM) to enable the movement of FFA from plasma into muscle cells; a pattern that mirrors the role of GLUT4 in glucose uptake. Both CD36 and GLUT4 sit in a vesicle in the cytoplasm and move to the PM upon stimuli reaching the muscle (e.g., insulin or contraction) [23,26]. It is suggested that high plasma insulin under IR leads to “permanent” translocation of CD36 to the PM, with consequent “open gate” scenario that leads to persistent increase in ectopic accumulation of fat [27]. On the contrary, there is less expression of GLUT4 in PM [28], with consequent reduced glucose uptake by the skeletal muscle, and development of chronic high blood glucose (hyperglycaemia).

Once inside the muscle cells, free fatty acids have three fates: β-oxidation in mitochondria, intramuscular triacylglycerol (TAG) or phospholipid synthesis, or synthesis of other lipid intermediates. Each of these pools play a role in determining the fate of intramuscular fat, but β-oxidation in mitochondria is of particular interest because it removes fat from muscles, circumventing the issue of ectopic lipid accumulation and IR. As Funai and Semekovich [29] beautifully put it, running water does not carry poison. Maintaining the intramyocellular lipid flux is another critical control point in skeletal muscle fatty acid metabolism which will impact glycemic control. Thus, mitochondrial biogenesis, a process by which cells increase their individual mitochondrial mass [30] to increase oxidative capacity in relation to stimuli, has assumed importance as a viable target for pharmaceutical approaches [31]. The key coactivator involved in stimuli-induced (hunger, exercise, etc.) mitochondrial biogenesis is PGC-1α (peroxisome proliferator-activated receptor-γ coactivator-1α). Recent reviews of the PGC family and mitochondrial biogenesis in relation to insulin resistance are available elsewhere [32,33]. Despite the biological logic behind the mitochondrial hypothesis for the genesis of skeletal muscle IR, it is still the subject of an on-going debate. For instance, Hoeks and Schrauwen [34] suggested that mitochondrial dysfunction (failure to mitigate accumulation of fat in skeletal muscle) is secondary to IR rather than a causative factor for IR genesis. It is not yet known whether the regulation (control switch) of which protein carrier translocates to PM to facilitate glucose and/or FFA uptake by the skeletal muscle is driven systemically or at the myocyte level. This is one of the critical control points that needs to be elucidated if we are to fully unravel the basis of skeletal muscle fuel selection and its contribution to insulin resistance and T2DM. In the following sections, we will summarise the evolving evidence linking different aspects of IMCL with IR.

## 3. Total Intramuscular Fat

The skeletal muscle is not a natural storage site for excess fat. When there is dietary lipid oversupply, the body responds by increasing the number and size of adipocytes [35]—the normal storage sites for excess fat. Storage of fats in non-fat-storage organs (liver, heart and skeletal muscle) ensues when the increase in adipocyte size and number fails to accommodate the excess dietary fat. The ensuing ectopic lipid accumulation, as a result of dietary lipid oversupply, is thought to be the driver of lipid-induced insulin resistance in the skeletal muscle [4,5,27]. Goodpaster et al. [36] were the first to report an increase in intramyocellular lipid in endurance athletes without consequent impaired glycemic control, which they termed “Athlete’s Paradox”. This was based on quantitative image analyses of muscle fibres stained using Oil Red O soluble dye which mainly stains TAG, and which yielded similar muscle lipid content (image area) between diabetic obese subjects and lean, exercise-trained subjects. It can be argued that this Athletes’ Paradox may have given impetus for researchers to look deeper than total IMCL. Consequently, the lipid-induced IR issue has now moved past total IMCL to the extent that “Athletes’ Paradox” could be considered a misnomer. A number of studies have shown that intramuscular TAG is more or less neutral (see review by Turner et al. [27]) with respect to skeletal muscle IR. The argument has moved from total IMCL to lipid classes/intermediates. In a follow up study, Goodpaster’s group also demonstrated exercise-induced increase in muscle TAG as well as decreases in DAG and ceramides [37]. In the last decade, ceramides and DAG have emerged as the main culprits for IR in skeletal muscle. Interestingly, Goodpaster’s group [37] found significant correlation between decrease in muscle ceramide content and increase in insulin sensitivity; but they did not find a correlation between decrease in muscle DAG and insulin sensitivity. This gives the perfect segue to look at DAG and ceramide as lipid intermediates that are associated with skeletal muscle IR.

## 4. DAG in Skeletal Muscle IR

In relation to IR, both DAG and ceramide accumulation in skeletal muscle belong to the lipotoxicity hypothesis [38] of IR genesis. The connection between ectopic accumulation of lipids and impaired glycemic control was widely known before Unger [38] coined the term lipotoxicity. The lipotoxicity (lipid metabolite) theory of IR essentially refers to excess inflow of FA into the skeletal muscle that overwhelms the use of FA in skeletal muscle for β-oxidation and triacylglycerol synthesis, leading to entry of excess FA into harmful non-oxidative pathways [39]. The DAG hypothesis of skeletal muscle IR gained significant impetus after the publication of Erion and Shulman [40], when they proposed it as a unifying and alternative hypothesis to the prevailing various hypotheses proposed to explain insulin resistance (inflammation in adipocytes, endoplasmic reticulum stress, increased reactive oxygen species and genetic alterations in insulin signalling). The mechanism proposed for DAG-induced IR is as follows [40]: increased plasma FA concentration leads to accumulation of intramyocellular acyl CoAs and DAG, which activates isoforms of protein kinase C (PKC), which, in turn, decrease the activities of PI3K and ISR-1 in the insulin signalling pathway for muscle glucose uptake (Figure 1).

As shown in Figure 1, nearly 95 years have passed since Banting and colleagues [8] discovered insulin. Although there have been constant discoveries and cloning of the genes involved in the insulin signalling cascade (Figure 1), the IR phenomenon remains complex, and there are wide variations of experimental evidence on how specific metabolites (e.g., DAG) influence the genesis of IR. For instance, there is discordance as to which PKC isoform is the major player in DAG-induced IR. Yu et al. [41] reported changes in protein kinase C- theta (PKC-θ) as a result of increased DAG accumulation following lipid infusion in male Wistar rats. Earlier, Griffin et al. [42] have also reported changes in PKC-θ in rats following lipid infusion, although they ascribed the change to activation of PKC-θ by high circulating FFA. On the other hand, lipid infusion in humans by Itani et al. [43] showed increased (three-fold) DAG accumulation had resulted in changes in the activities of PKC-beta II (PKC-βII) and PKC-delta (PKC-δ). Interestingly, the authors also reported a 70% decreased in the inhibitor of NFkβ (Ikβ-α), which is significant with respect to the link between IR and inflammatory status. Furthermore, they found no change in the ceramide content of skeletal muscle. Recently, Li et al. [44] conducted an in-depth study of PKC-δ in mice that had undergone muscle-specific deletion of PKC-δ. They found that PKC-δ levels increased with age and the deletion of PKC-δ in muscle improved whole body insulin sensitivity and muscle IR from six to seven months of age onwards. Their study showed age-related increase in the significance of PKC-δ in regulating insulin sensitivity, which may explain some of the variations between studies. Similarly, Szendroedi et al. [45] conducted a detailed investigation of the association between PKC-θ and muscle DAG using serial muscle biopsies of lean, obese and obese-diabetic human subjects before and during lipid infusion. In all cases, they found that total and cytosolic DAG accumulation was associated with PKC-θ activation and decrease in insulin signalling in skeletal muscle. In summary, DAG-induced change in insulin signalling in skeletal muscle appears to be through activation of PKC, but the specific PKC isoform involved seems to be different between rodents and humans. 

In addition to the discordance in the type of PKC isoforms reported in relation to DAG and IR, not all changes in muscle DAG have been associated with skeletal muscle IR (Table 2 and Table 3). Overall, the in vitro cell culture results (Table 2) generally show relatively consistent association between muscle DAG and muscle IR. The responses in the in vivo animal model systems (Table 3) are blunted or non-existent. This is not surprising as the in vitro cultures are usually single fatty acid incubations, while in the in vivo systems, myotubes are faced with a milieu of fatty acids and other nutrients and their metabolites. The starkest discrepancy is that reported by Selathurai et al. [46], where a 200% increase in muscle DAG (Table 3) following muscle-specific knockout of phosphoethanolamine cytidylytransferase (ECT) failed to induce insulin resistance in the KO mice. The authors noted an unexpected increase in muscle mitochondrial biogenesis in ECT-deficient mice, which may have blunted the impact of marked increase in muscle DAG. However, the deletion of ECT also caused significant changes to the phospholipid pool and the exact mechanism that explains the observed absence of IR in muscle that exhibited 200% increase in DAG remains unresolved. The latest evidence suggests that even within a particular lipid intermediate, such as DAG, the regio- or stereo-isomers of the lipid class may be important in determining its potency in inducing skeletal muscle IR. For instance, Szendroedi et al. [47] reported that DAG species that contained C16:0, C18:0, C18:1, C18:2 or C20:4 FA showed the strongest relationship with PKC-θ activation and IR in obese and T2DM individuals. This concurs with Ritter et al. [48] who also suggested both the DAG species and its subcellular localisation (cytosol versus membrane) are important considerations with regards to PKC-θ activation by DAG.

In summary, there is a link between muscle DAG and IR in skeletal muscle. This link seems to be mediated through the activation of the PKC pathway, although the particular PKC isoform involved remains to be confirmed. There is emerging evidence that the DAG species in muscle is also an important consideration in addition to total muscle DAG. As far back as 1979, Takai et al. [49] suggested that unsaturated diacylglycerol is a possible messenger for the activation of calcium-activated, phospholipid-dependent protein kinase system. The debate on diacylglycerol as a physiological mediator of hormone action through the PKC system seems to have come a full circle since the initial work by Nishizuka [50]. The discrepancy in the literature in relation to DAG and IR may be a function of the study model (cell culture, animal model species, knockout animal models, or human subjects), the species of DAG generated by particular lipid interventions, or the duration of lipid intervention (acute versus long-term). As Turner et al.’s [51] work showed, high-fat diet induced loss of glycemic control can manifest itself within days without sufficient time for change in muscle lipid profile. The balance of evidence suggests that there is a link between intramyocellular DAG and IR in skeletal muscle. As Hannun and Obeid [52] pointed out, the original concept that a lipid can regulate cell signalling was cemented by the observation of direct activation of PKC by DAG, which led to the current era of bioactive lipid research. The expanding use of lipidomics will most likely establish which DAG species are responsible for generating the PKC isoform/s that is vitally linked with IR in in vivo human clinical settings, and pave the way for pharmacological interventions that will arrest the progression of DAG-induced skeletal muscle IR to T2DM.

## 5. Ceramides in Skeletal Muscle IR

**Figure 2:**
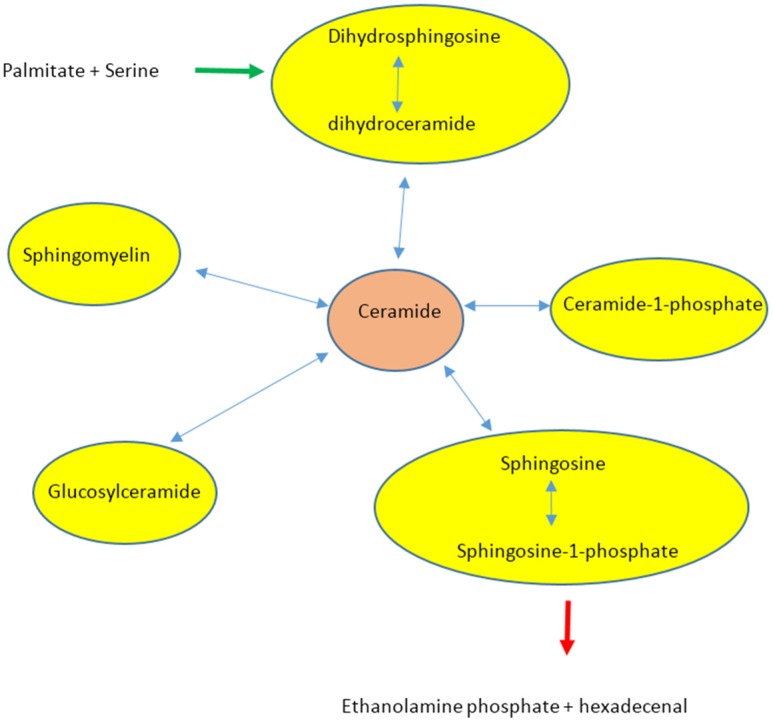
A brief schematic display of the interconversions of ceramide and other sphingolipids. Adapted from Hannun and Obeid [52]. Full enzymatic details can be found elsewhere [52].

A comprehensive and detailed review of sphingoid metabolism and the role of sphingolipids in cell signalling is presented by Hannun and Obeid [52]. Sphingolipid metabolism is very complex and there are estimated to be 4000 to 60,000 sphingolipid mediators [55]. It is even suggested that there is a need for separate “omics” for sphingolipids–sphingolipidome [52,55]. The major bioactive sphingolipids are: sphingosine, sphingosine-1-phosphate (S1P), ceramides, ceramide-1-phosphate (C1P), glucosylceramide, lyso-sphingomyelin and dihydroceramide [52]. A schematic representation of the sphingolipid biosynthetic pathway is shown in Figure 2. As Hannun and Obeid [52] pointed out, ceramide is the metabolic hub because it occupies the central position in sphingolipid biosynthesis and catabolism. The entry point for FA in Figure 2 is the de novo synthesis of dihydroceramide from palmitate and serine (catalysed by serine palmitoyl-transferase, SPT), which is converted to dihydroceramide that is desaturated to ceramide [52]. Other inputs to the ceramide pool are from sphingosine by ceramide synthase, from sphingomyelin by sphingomyelinase, from ceramide-1-phosphate by phosphatase and from glucosylceramide by glucosyl ceramidase. The exit point from sphingolipid pool is through conversion of ceramide to sphingosine followed by S-1-P, which is then catabolised to ethanolamine phosphate and hexadecenal involving S1P lyase. Clearly, ceramide metabolism involves complex enzymatic interconversions with other sphingolipids. Ceramide synthase alone has six variants numbered accordingly. To complicate matters further, Hannun and Obeid [52] suggested that the bioactivity of sphingolipid intermediates may vary depending on their subcellular localisations (cytosol versus PM), even including whether localised to the internal or external leaf of the PM. Taking this into consideration, it is not surprising that there are reports where skeletal muscle IR had been detected without increase in total ceramide or increased total ceramide has been detected without skeletal muscle IR.

With respect to ceramide and IR, Summers and colleagues have contributed a series of experiments [56,57,58,59] and review articles [60,61,62,63]. Muscle cell culture studies involving incubation of myotubes in palmitate have shown that the development of IR was strongly related to increased ceramide accumulation [56,57,58,59]. The initial confirmation of this hypothesis was achieved using pharmacological inhibitors of de novo ceramide synthesis in prediabetic obese rats [64]. Blocking the synthesis of ceramides prevented the development of insulin resistance or led to recovery of glycemic control when it was used as an intervention. Myriocin has been the commonly used agent to block ceramide synthesis in skeletal muscle [65] and other tissues. The mechanism by which ceramide inhibits insulin-stimulated glucose uptake by the muscle and leads to IR is thought to be through its effect on stimulation of Akt/PKB (Figure 1). The effectors for this stimulation are protein phosphatase 2A (PP2A) and PKC-zeta (PKC-ζ) [62]. 

As in the DAG story, not all incidences of impaired glycemic control have been associated with increased ceramide in IMCL [53,66,67,68]. The reports by Skovbro et al. [66], Helge et al. [67] and Dubé et al. [68] are of particular note because they were based on skeletal muscle samples from human subjects. Similar to the DAG story, it is now emerging that the focus may need to be on the nature of ceramide species involved rather than the total ceramide in IMCL. In 2014, two animal model studies [69,70] were published identifying C16:0 ceramide as the sphingolipid that mediates the pathophysiology of IR in liver. The importance of ceramide species in skeletal muscle IR has recently been published by Bergman et al. [71]. Unlike the liver IR in animal model studies, their human volunteer studies showed that muscle C18:0 ceramide was inversely related to insulin sensitivity in a study involving obese subjects, endurance-trained athletes, and subjects with T2DM. Interestingly, the authors reported other C18:0 sphingolipid molecules, dihydroceramide and glucosylceramide, were also significantly correlated with obesity and IR. Driving the importance of sphingolipid species, the authors did not find significant changes in total ceramide, total dihydroceramide or total glucosylceramide. It remains to be determined if the difference in the ceramide species identified relates to the mice-human contrast or the liver-muscle contrast between the studies. The results from Bergman et al. [71] are in agreement with prior report by De La Maza et al. [72], who found strong association between ceramide species, not total IMCL ceramide, and glucose intolerance when comparing male volunteers of contrasting BMI (BMI < 25 versus BMI > 25). However, the ceramide species their study identified as relevant to glucose intolerance were the longer chains C20:1, C22:0 and C22:1. To add further complication to the picture, Kasumov et al. [73] noted that exercise-related improvement in insulin sensitivity was linked to plasma C14:0 ceramide in a study that compared obese-NGT (normal glucose tolerance) and obese-T2DM volunteers. In summary, there is now evidence that specific species of ceramides are of greater importance than the total ceramide in IMCL. It is not yet clear if there are key ceramide species that are linked to loss of insulin sensitivity and development of IR for each species (rodents versus humans), or tissue (liver versus muscle). This will become apparent with increasing use of lipidomics. It is also worth exploring the relationship between the tissue level concentration of ceramide species identified as being linked to IR (e.g., C18:0 ceramide), and its systemic concentration (plasma).

## 6. Conclusions

As shown in Table 4, the search for lipid bioactive responsible for lipid-induced IR in skeletal muscle can be divided into three phases. There is the initial phase that identified close association between increase in total IMCL and IR. Different glycemic control outcomes for people with similarly high IMCL content led to the realisation that particular lipid intermediates (DAG and ceramide), rather than total IMCL, was what is important with respect to the genesis of IR. This can be considered Phase II. The third phase relates to the emergence of normal glycemic control despite high DAG/ceramide content in skeletal muscle, which led to the identification of species of DAG and ceramide using lipidomics. The latter has provided some insights into molecular mechanisms underpinning the links between the accumulation of specific DAG and ceramides species and the presence of skeletal muscle IR. Although the expansion of the application of lipidomics has provided some explanation for the inconsistencies between different published results, it has also thrown up some unforeseen complications leading to the possibility that the lipid intermediate species responsible for IR in rodents may be different from that which influences IR genesis in humans. It is now evident that particular DAG and ceramide species are more important than the total IMCL ceramide or DAG content. In this regard, the lipidomics approach has taken the lipid-induced IR issue a step further. There is strong evidence that both DAG and ceramides exert their influence through activation of the PKC pathway, although different PKC isoforms are indicated. The holy grail of unravelling the genesis of IR in skeletal muscle still has some way to go. It still remains to be determined if an association between a particular DAG/ceramide species and IR is a universal outcome for a species (rodent/human), for a tissue (adipose versus liver versus muscle), is situation specific, or modified by the epigenetics of the person/animal. Even with full knowledge of the lipidomics profile of IR and non-IR subjects, influencing the enzyme systems in lipid metabolism, such as sphingolipid metabolism, towards a desirable phenotype/profile is fraught with difficulties because the enzyme systems are intimately interconnected, which Hannun and Obeid [52] termed the difficulty of the “ripple effect”. It is worth mentioning at this juncture that only a tiny fraction of the lipid pool in a muscle is DAG or ceramide. With continuing progress in cloning of the key enzymes involved in sphingolipid metabolism (all of the six ceramide synthases have been cloned), the capacity to block particular ceramide species synthesis will become easier, at least experimentally.

Generally speaking, the whole IR issue requires identifying which factors have primary roles and which factors play secondary roles in the genesis of skeletal muscle IR. It is worth stressing that dietary FA supply is at the core of lipid-induced IR in skeletal muscle. It is the one lever that can be dialled up/down to regulate the flow of lipid intermediates into organs not intended for lipid storage. In a free living and affluent society, controlling this primary factor has proven to be an impossible task as a population-wide nutrition strategy for good public health. Hence, much research is devoted to secondary and tertiary factors in the genesis of IR. For instance, there is an emerging interest in the role of microbiota and IR [74], the key mediator identified being Toll-like receptor-4 (TLR4). This will continue to provide a number of insights as to how the gut microbiota links to energy utilisation and the development of IR. It is worth posing this question: Does searching the ideal lipidomic, sphingolipidomic and microbiota profile (that prevents lipid-induced IR) go against the principle of Occam’s razor? Our understanding of the lipid intermediates and their mode of action has increased over the last decade. At present, these advances have not yielded a clear alternative to reducing dietary fat supply and maintaining the intramuscular fat pool in a state of flux through exercise and active life style.

## Figures and Tables

**Table 1:** Sites of activities of fatty acid transport proteins (extracted from Kazantzis and Stahl [24]).

Transporter	Major Site/Organ
FATP1	White adipose tissue (WAT), brown adipose tissue (BAT), *skeletal muscle*, heart (lesser extent: pancreas, lung, kidney and brain)
FATP2	Kidney and liver
FATP3	Lung, liver, pancreas and endothelial cells of capillaries in many organs
FATP4	Broadly distributed; heart, liver, kidney, *skeletal muscle*, brain, skin, and endothelial cells. The predominant FATP in small intestines
FATP5	A liver-specific protein
FATP6	Exclusive to heart

**Table 2:** Intramyocellular lipid changes and consequent impact on muscle glucose uptake or insulin resistance in studies where myotubes were cultured with different fatty acid mixtures.

Model	Fat Intervention/s	Muscle Lipid Change	Glucose Uptake/Insulin Resistance	Reference
L6 myotubes	PA, LA, DHA at 0.4 mmol/L	**Muscle DAG:**	**Muscle glucose uptake:**	[45]
			PA: ↓66%	
		↑16-fold PA	PA + DHA: 20↑	
		↓45% DHA	PA + LA: 55%↑	
		↓65% LA	Myriocin: 2.0 to 2.5-fold↑	
		**Muscle Ceramide (in presence of PA):**	Myriocin inhibits ceramide synthesis	
		↑2.5-fold by DHA	DHA increases hydrolysis of sphingomyelin	
		↑1.9-fold by LA		
L6 myotubes	Palmitic acid (Palmitate), Linoleic acid (Linoleate)	**Muscle DAG:**	**Muscle glucose uptake:**	[53]
		↑5-fold PA		
		No change by LA		
	0.5 mmol/L	**Muscle Ceramide:**	↓by PA	
		↑2-fold by PA	Unaffected by LA	
		No change by LA		
C12C12 myotubes	Normal FA mixture (40% SFA), high SFA FA mixture (60% SFA) & 100% Palmitic acid	**Muscle DAG:**	Akt phosphorylation impaired (*p* < 0.05) by 100% PA.	[54]
	Doses: 0.1, 0.2, 0.4 or 0.8 mmol/L	DAG accumulation unchanged with increasing dose of the two mixtures.	Modest impact of the two mixtures. No difference between 40% & 60% SFA.	
		DAG accumulation increased with increasing dose of 100% PALM.		
Vastus lateralis biopsies (Obese non-diabetic men & women)	Normal FA mixture (40% SFA), high SFA FA mixture (60% SFA) & 100% Palmitic acid	**Muscle DAG:**	Akt phosphorylation impaired (*p* > 0.05) by 100% PA.	[54]
	Dose: 0.4 mmol/L	100% PALM increased DAG over no FA control as well as the two FA mixtures.	Modest impact of the two mixtures. No difference between 40% & 60% SFA.	

DAG, diacylglycerol; DHA, docosahexaenoic acid; FA, fatty acid; LA, linoleic acid; PA, palmitic acid; SFA, saturated fatty acid.

**Table 3:** Intramyocellular lipid changes and consequent impact on muscle glucose uptake or insulin resistance from *in vivo* animal model feeding studies.

Experimental Background	Dietary Fat Intervention/s	Muscle Lipid Change	Glucose Uptake/Insulin Resistance	Reference
Muscle specific ECT Knock out mice, 4 weeks duration	5% Cal from fat	DAG: 200%↑	No change detected	[46]
	Control versus ECT KO			
	18 week old male mice			
	42% Cal from fat	DAG: 200%↑	No change detected	[46]
	Control versus ECT KO			
	6-week old male mice			
Sprague–Dawley rats, male, 95–110 g, 8 weeks duration	**Control**: 15.7% fat	Muscle DAG: (SFA = PUFA) > Control	HOMA-IR: SFA > Control PUFA < Control	[53]
	**High SFA**: 52.8% fat from lard and coconut oil.	(SFA = PUFA) > Control		
	**High PUFA**: 52.8% fat from safflower oil			
C57Bl/6 mice, 8–12 weeks old	Std chow, 5% energy from fat versus HFD (45% en from fat), endpoints at 1, 3, 6 and 16 weeks	At 3 weeks, muscle DAG increased over control.	At 3 weeks, muscle IR detected in HFD group	[51]
		At 3 weeks, muscle ceramide 18:0 increased over control.		

DAG, diacylglycerol; ECT-KO, phosphoethanolamine cytidylyltransferase knock out; HFD, high-fat diet; HOMA-IR, homeostasis model assessment- insulin resistance; IR, insulin resistance; PUFA, polyunsaturated fatty acids; SFA, saturated fatty acids.

**Table 4:** Summary of progress in identifying skeletal muscle fat contents responsible for lipid-induced insulin resistance in skeletal muscle.

Phase	Hypothesis	Size of Bioactive in Muscle	Main Suggested Mechanism
Initial association studies	Total IMCL	1–5 g/100 g muscle	General interference with glucose metabolism
Detailed lipid metabolism studies	Total DAG and/or Ceramide	pg/g muscle	Interference with insulin signaling (PKC pathway)
Lipidomics	Specific DAG and/or ceramide species	A fraction of pg/g muscle	Interference with insulin signaling (PKC pathway)

DAG, diacylglycerol; IMCL, intramyocellular lipid; PKC, protein kinase C.
